# PiMP my metabolome: an integrated, web-based tool for LC-MS metabolomics data

**DOI:** 10.1093/bioinformatics/btx499

**Published:** 2017-08-14

**Authors:** Yoann Gloaguen, Fraser Morton, Rónán Daly, Ross Gurden, Simon Rogers, Joe Wandy, David Wilson, Michael Barrett, Karl Burgess

**Affiliations:** 1 Institute of Infection, Immunity and Inflammation, Glasgow Polyomics, University of Glasgow, Glasgow, UK; 2 Centre for Cell Engineering, University of Glasgow, Glasgow, UK; 3 School of Computing Science, University of Glasgow, Glasgow, UK; 4 Wellcome Trust Centre for Molecular Parasitology, Institute of Infection, Immunity and Inflammation, University of Glasgow, Glasgow, UK

## Abstract

**Summary:**

The Polyomics integrated Metabolomics Pipeline (PiMP) fulfils an unmet need in metabolomics data analysis. PiMP offers automated and user-friendly analysis from mass spectrometry data acquisition to biological interpretation. Our key innovations are the Summary Page, which provides a simple overview of the experiment in the format of a scientific paper, containing the key findings of the experiment along with associated metadata; and the Metabolite Page, which provides a list of each metabolite accompanied by ‘evidence cards’, which provide a variety of criteria behind metabolite annotation including peak shapes, intensities in different sample groups and database information.

**Availability and implementation:**

PiMP is available at http://polyomics.mvls.gla.ac.uk, and access is freely available on request. 50 GB of space is allocated for data storage, with unrestricted number of samples and analyses per user. Source code is available at https://github.com/RonanDaly/pimp and licensed under the GPL.

**Supplementary information:**

Supplementary data are available at *Bioinformatics* online.

## 1 Introduction

Metabolomics aims to catalogue and quantify the complete small molecule complement of a biological system (Oliver *et al.*, 1998). Liquid chromatography–mass spectrometry (LCMS) is now the most common analytical technique used to generate metabolomics data, and the methodology is capable of detecting hundreds to thousands of metabolites from a single sample. While the analytical platforms available are well developed (Dunn *et al.*, 2011), methods for linking the detected features, through identification of metabolites, to the interpretation of biological context are poorly developed. Annotation tools, pathway mapping tools and statistical tools often consist of individual functions or packages that must be invoked separately, requiring modification of data formats. Here we present a comprehensive and integrated web enabled pipeline: the Polyomics integrated Metabolomics Pipeline (PiMP). Through extensive interaction with end users, we have developed a workflow aimed at researchers with a modest background understanding of metabolomics and biochemistry, but with a need to garner vital information now available through MS-based metabolomics experimentation. The processing of metabolomics data in PiMP is presented as an assisted pipeline consisting of five sequential tasks: (i) project administration, (ii) data upload, (iii) quality control, (iv) analysis parameters and (v) data interpretation. This assisted pipeline provides guidance to users analysing metabolomics data without necessarily having significant prior knowledge pertaining to metabolomics workflows or even biochemistry. Results of experiments are shown using a tabbed display system, providing access to different contexts in which the data can be evaluated. These are: the **summary** tab; the **metabolite** tab and the **pathways** tab. Comparing the three major online platforms available (XCMS Online (Tautenhahn *et al.*, 2012), Workflow4metabolomics (W4M) (Giacomoni *et al.*, 2015) and MetaboAnalyst (Xia *et al.*, 2012), W4M provides a user-friendly front end to XCMS, but is limited to default visualizations and provides no biological inference for the results and MetaboAnalyst provides extensive statistical tools and interpretation, but lacks the contextual design of PiMP. XCMS Online provides the majority of PiMPs features, but is lacking the modular design and open development model of PiMP. Indeed a third party module has already been developed interfacing PiMP with MetExplore (Cottret *et al.*, 2010).

## 2 Materials and methods

The user-interface of PiMP is written in common Web standards and accessible from any modern Web browser. Where possible, portions of the user interface are selectively retrieved and updated via asynchronous Javascript to improve perceived response time. The back-end of the PiMP web application is written in Python using Django. Django provides an abstraction layer to the database using object-relational mapping techniques. PiMP uses MySQL to store the data in a relational database. The analysis components in PiMP are implemented as an R pipeline based around XCMS (Colin A. Smith *et al.*, 2006) for the feature detection and mzMatch.R (Scheltema *et al.*, 2011) for common metabolomics data pre-processing tasks (*e.g.* alignment, batch correction and identification). All these components are gathered in a Docker container for easy deployment, both locally and on a shared server. Analysis results are returned to the web application via a PiMP-specific XML format, allowing for the possibility of a new computational pipeline to be used in place of the current pipeline, provided that the same output schema is maintained. From the user interface, analysis results can also be exported into text files for processing outside PiMP.

## 3 Using PiMP

**Fig. 1:**
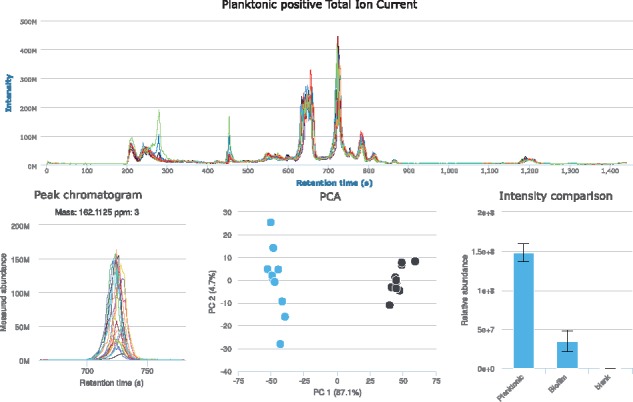
Overview of the Polyomics integrated Metabolomics Pipeline—sample graphs from results tabs (Color version of this figure is available at *Bioinformatics* online.)

Please refer to the Supplementary Information for a comprehensive user manual. PiMP’s project administration interface allows the user to define the experimental design, specify metadata (e.g. the study organism or specific tissue studied), and to share the project with collaborators with a chosen level of permission. The data can be uploaded by simple drag and drop. Visualization tools then allow the user to assess the quality of the data by accessing total ion chromatograms (TICs), viewing and curating raw data or looking for specific features/compounds including internal or external standards. The pipeline supports pairwise and combinatorial comparisons. Default parameters for XCMS and mzMatch are suggested to the user for data analysis, but alternatives are readily available via drag-down menus. Results are presented in a unified data exploration environment, organized into tabs (Fig. 1). The **summary** tab contains the key metadata for the experiment, along with TICs allowing basic visualization of the reproducibility of each sample group. Principal component analysis plots are also provided for the dataset as a means of rapid quality control for the data. Experimental comparisons are then displayed with significant quantitative differences highlighted using histograms and a volcano plot, allowing metabolites corresponding to the largest changes between experimental conditions to be analysed. While for biomarker discovery applications, this may be sufficient as a starting point, biochemical context is often key to interpretation of a dataset. The **metabolites** tab provides a powerful means of understanding the biochemical changes observed in an experiment. It is based around the concept of ‘evidence’ for a particular metabolite. Any metabolite for which evidence for its existence is available is presented. This evidence, along with quantitative information, peak chromatograms, pathway information and structures are presented in a sidebar. To provide context for metabolites, filtering based on pathway and superpathway is available from the toolbar, and with the colour-coding for fold change between groups, the quantitative modulation of a given pathway can readily be assessed. The **pathways** tab then provides a summary of the detected pathways, and the sidebar allows direct access to the KEGG map associated with a chosen pathway (Kanehisa *et al.*, 2014). Once the map has been generated, comparisons can be overlaid on the map, providing a colour-coded overview of metabolism, in the same way as other applications (Leader *et al.*, 2011; Suhre and Schmitt-Kopplin, 2008). Two other tabs are available: the **comparison** tab and the **peaks** tab, which contain the raw peak data. Due to the number of unknown compounds detected, even after filtering for adducts and fragments, these are available for projects where information on all peaks detected is needed, irrespective of our ability to annotate them as putative metabolites.

## Supplementary Material

Supplementary Data
