# Supplementary material for: PiMP my metabolome: an integrated, web-based tool for LC-MS metabolomics data
Source: Bioinformatics. 2017 Aug 14;33(24):4007–9. doi: 10.1093/bioinformatics/btx499 (PMC5860087; doi:10.1093/bioinformatics/btx499)
Supplement: Supplementary Data [file btx499_pimp_user_guide.pdf]

User guide

PiMP

Polyomics integrated Metabolomics Pipeline
v. 1.0

University of Glasgow

Glasgow Polyomics
www.polyomics.gla.ac.uk

Getting started

Please Note

For space and portability reasons, PiMP relies on centroided single polarity data in the MzXML format. If you are unable to convert your data into MzXML, please look at the msconvert tool guide.

PiMP supports all latest web browser, however features are primarily developed using Google Chrome, we strongly advise users to use this web browser for optimal experience. Chrome can be downloaded here.

Logging in to PiMP:

To log in to PiMP, go to: PiMP Login page

Once there, please input your username and password. If you have not been provided with a username and password, please contact the PiMP administrator. Once you hit enter, or click 'Submit', you should find yourself in your profile page. From here you can see some general information about your projects, collaborators and the amount of storage you have used.

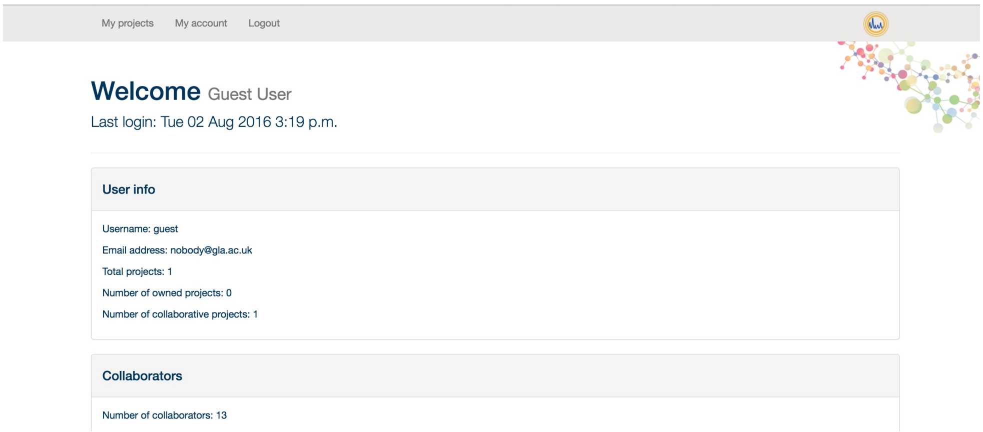

From here, you can click on 'My Projects' to create a new project and start your analysis.

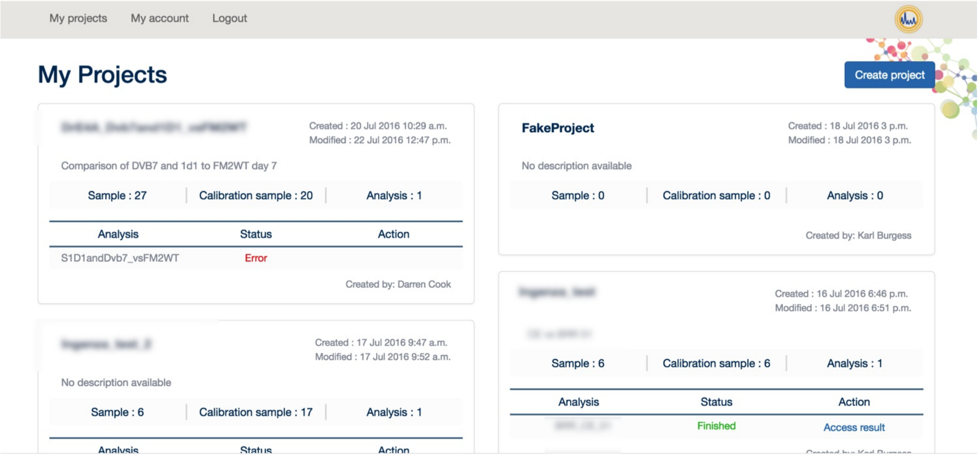

Prerequisites

To analyse your data, you first need:

1. Your data in MzXML format
2. Your experimental design

Once you have these things available, please click on ‘Create Project’.

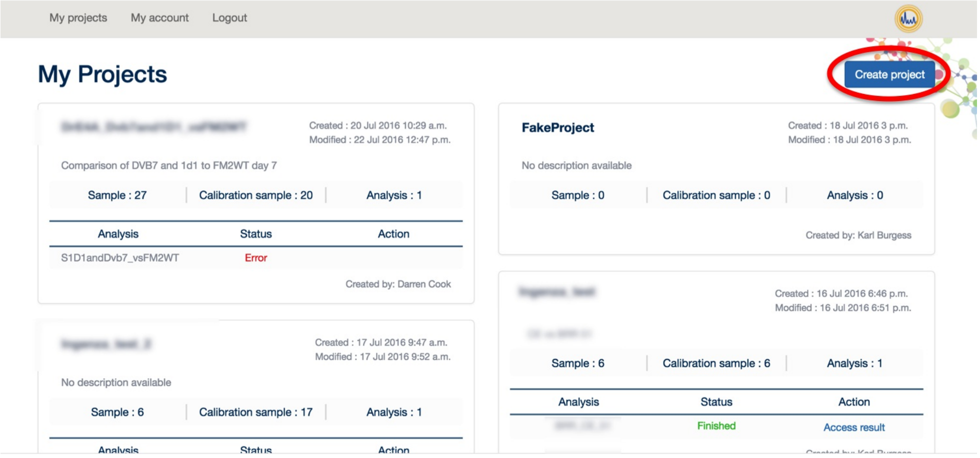

Create Project:

To create your project, first give it a title, then a description. The description is useful as it allows you to tag your projects so they are easily browsable. Then click 'Create project'.

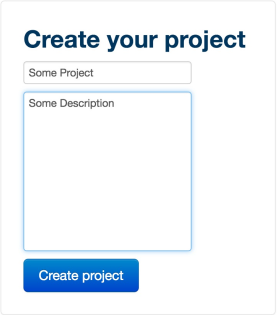

Project Administration:

In ‘Project Administration’, you can add users to your project (via the ‘add users’ button), edit the title of your project or change the description (via the ‘edit title’ and ‘edit’ buttons respectively.

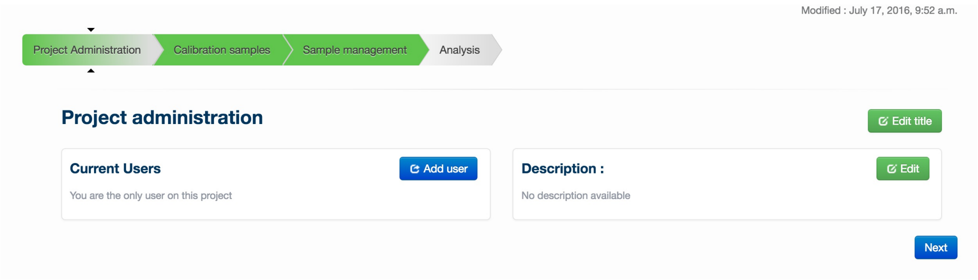

Once you have performed any desired administration in this page, enter calibration samples by clicking on the ‘Calibration Samples’ tab.

Calibration Samples:

To start creating a metabolomics experiment, you can first provide a group of supporting data files. Note that files are not necessary for the analysis to run. These supporting files consist of blanks, standards files and pooled/QC files. The blanks and pooled/QCs must be in MzXML format and the standards files must be in CSV format using the default Polyomics standards mixes (click here for downloadable examples; Standards 1, Standards 2, Standards 3). MzXML files must be uploaded in pairs: both a positive ionization mode and negative ionization mode version of each file. The pair of files must have the same name. Once a pair is uploaded, this is denoted by + - symbols next to the filename.

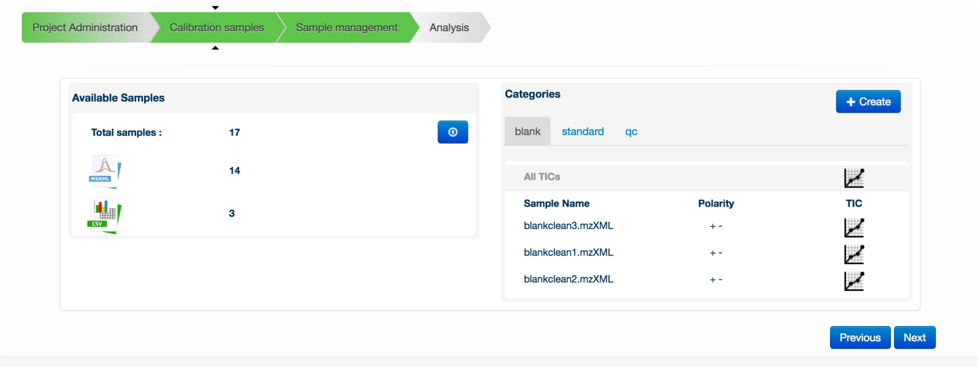

To upload any of the files, click on the blue button with an up arrow in the Available Samples box. A new window will appear allowing you to drag and drop your files for upload.

Upload File (calibration data):

Simply drag and drop the blanks and pooled/QC MzXML files and standards files in csv format into the box below, then click the green ‘start upload’ button.

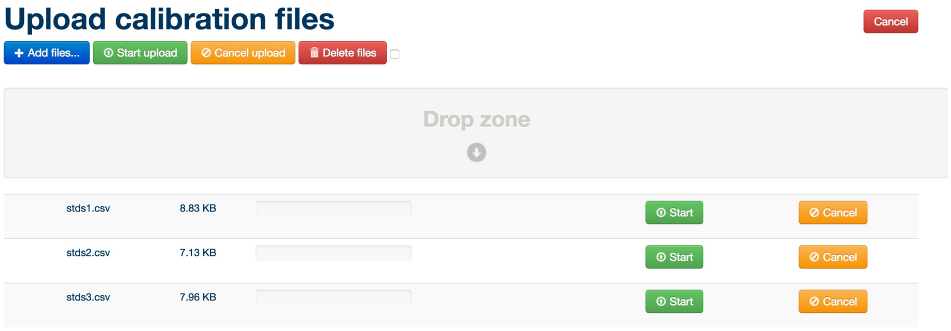

MzXML files must be uploaded in pairs: both a positive ionization mode and negative ionization mode version of each file. The pair of files must have the same name. Once a pair is uploaded, this is denoted by + - symbols next to the filename.

Once your samples are uploaded, assign them to their appropriate categories (either standards, blank or QC).

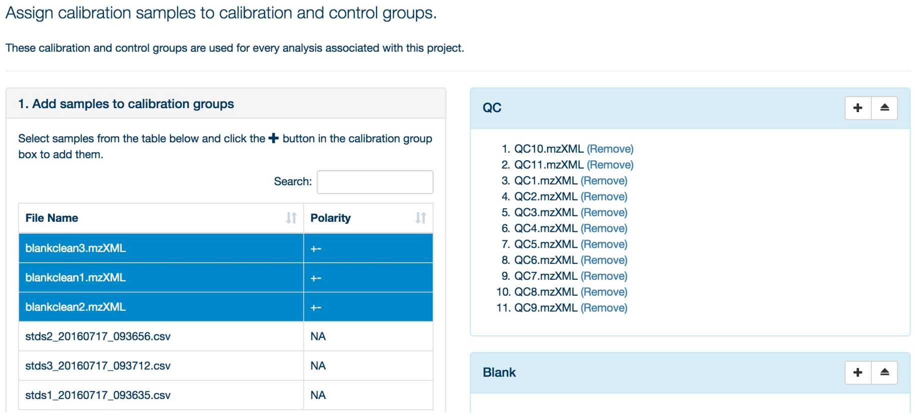

To assign your samples to groups, highlight the samples in that group and then click the '+' symbol next to the appropriate group title.

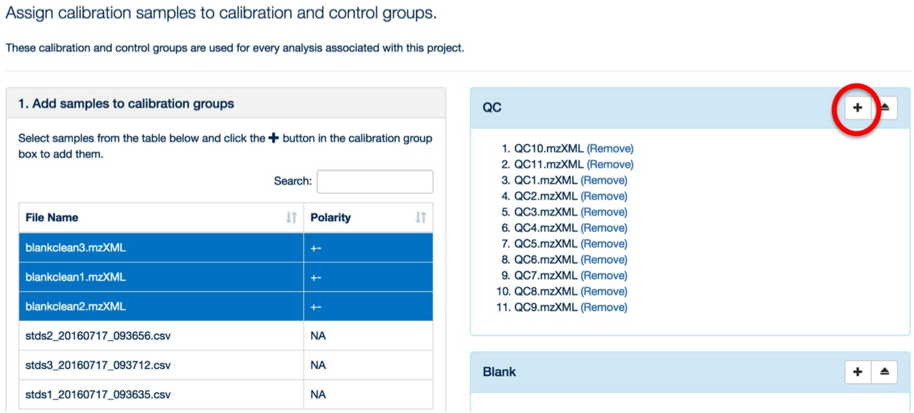

You can use the 'search' box to free text filter the file list.

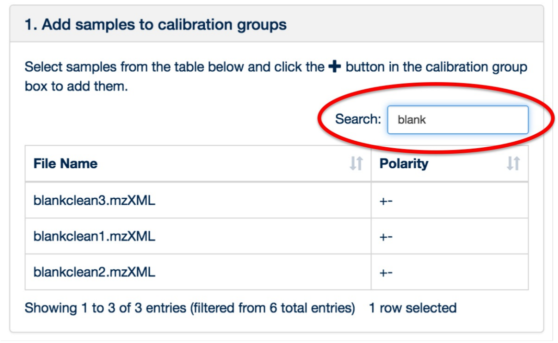

Once all of the files have been uploaded and assigned, click the ‘Sample Management’ tab.

Sample Management:

To begin uploading files, click the blue ‘upload files’ button in the ‘Samples’ box. Once your samples are uploaded, you can apply an experimental design by clicking on the ‘Create’ button.

> *Tips*
>
> - Use the peak discovery tool to seach for specific compounds or internal standards in your samples before starting the analysis. To access it, click on 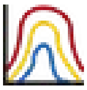

Experimental Design:

Currently, PiMP supports experiments defined by discrete categories. Thus experiments must be defined in terms of conditions: wild type, mutant, time 0, time 30, no drug, low dose, high dose, etc. You can have as many conditions as you like, although bear in mind that a large number of conditions in a single experiment are hard to visualize effectively – you might be better to create multiple experiments. Once you have defined your conditions, click ‘Next’.

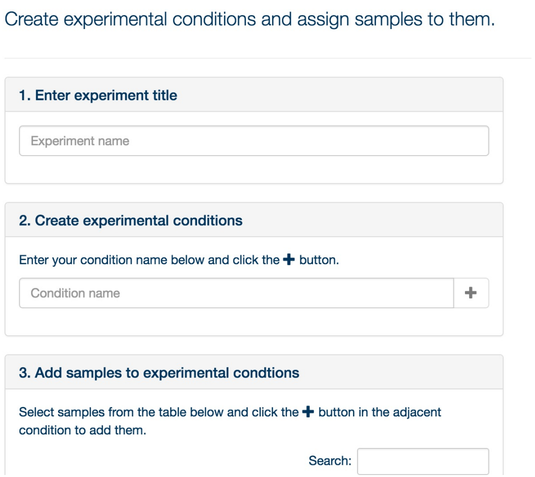

Assign Samples:

You can now drag and drop the files into the conditions you have specified. Once you have completed your assignment, click ‘Submit’, and click on the ‘Analysis’ tab.

Analysis:

Once you have defined a project and set up the calibration and experimental samples, you can create a new analysis to be submitted to the server, you must click on the ‘+ Create’ button.

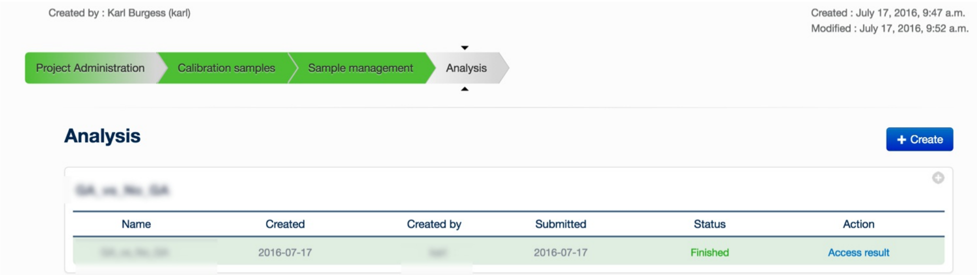

You may provide an experiment name, and define the comparisons to be made. A comparison is simply a statistical comparison between two experimental groups, e.g. wild type vs mutant, or time 90 vs time 0. You can create as many comparisons as you like: click ‘Add Comparison’ to add another to the list for this experiment. It is also possible to change the default parameters to XCMS and mzMatch, by clicking the Change Parameters tab. When you have created the comparisons that you are interested in, click ‘Save'.

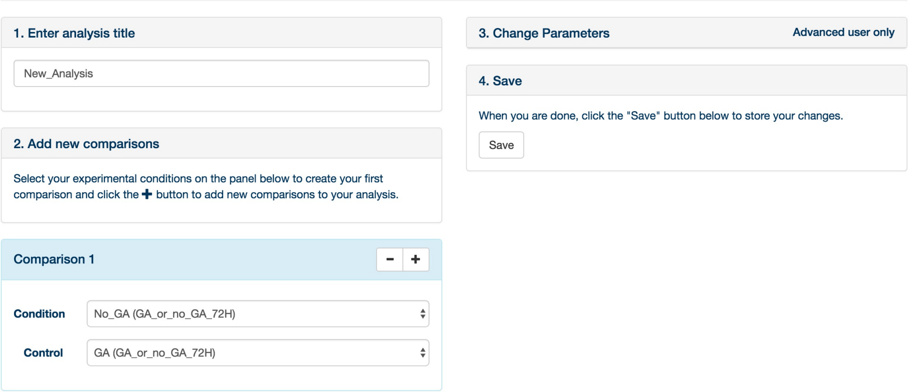

You are returned to the ‘Analysis’ tab. Your newly created analysis will be at the bottom of the list of current analyses. Simply click ‘Submit analysis’ to submit the analysis to the server. A lot of computational analysis is now performed on the data – please refer to this paper for a description of the analytical process. When the analysis is finished, you can click on the ‘access result’ button, which will bring you to the data exploration environment.

Analysis Performance

It might be wondered at what speed the analysis proceeds at. The following plot shows performance at sample sizes of 6, 12, 24, 48, 96 and 132. These runs were completed on a computer with two Intel Xeon E5-2698 CPUs running at 2.3 Ghz, totalling 32 cores with two Hyper-threads per core and 256 GB of

RAM. As can be seen, the runtime for the analysis in this case is roughly linear, with a rate of roughly 10 samples per hour.

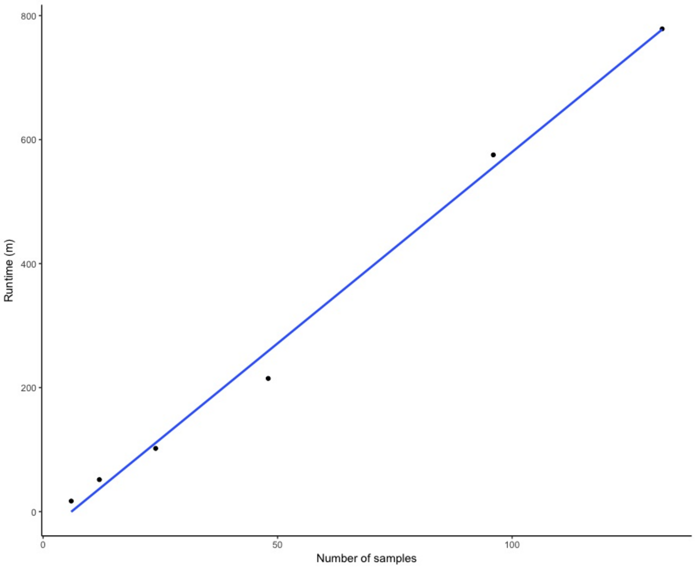

Results

Once your analysis is completed, it will appear in your 'My Projects' page with a green 'Finished' label in your project card.

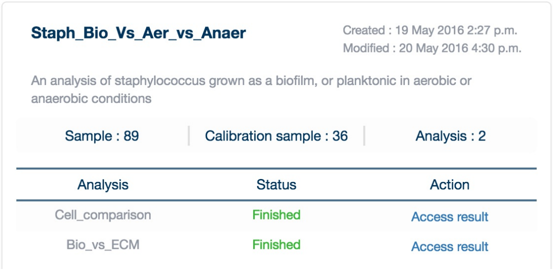

Click on 'Access Result' to load the data into your browser. A loading screen will appear (please be patient, many projects are quite large!).

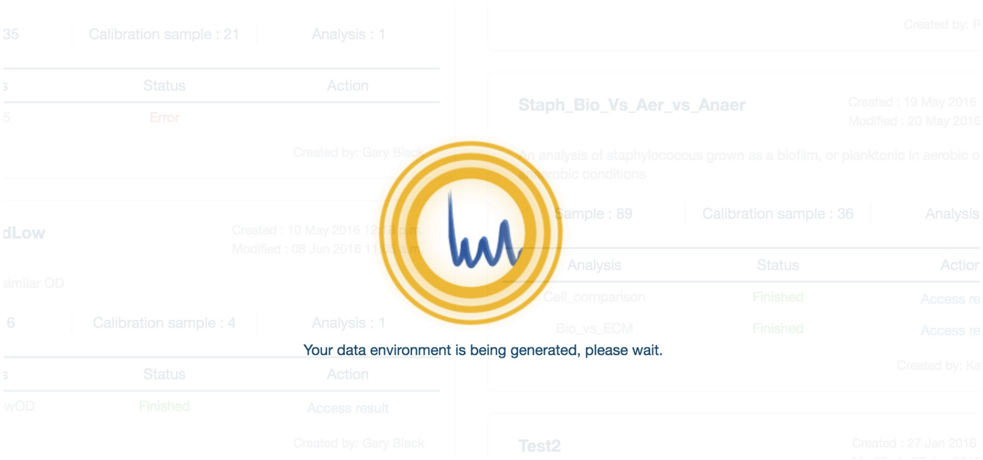

Data Environment:

The first page that loads once you select a completed analysis is the Summary Report.

The PiMP Summary Report displays a summary of the key information from the study. ‘Study’ describes the experimental design that has been chosen, the table shows the groups and number of replicates for each group.

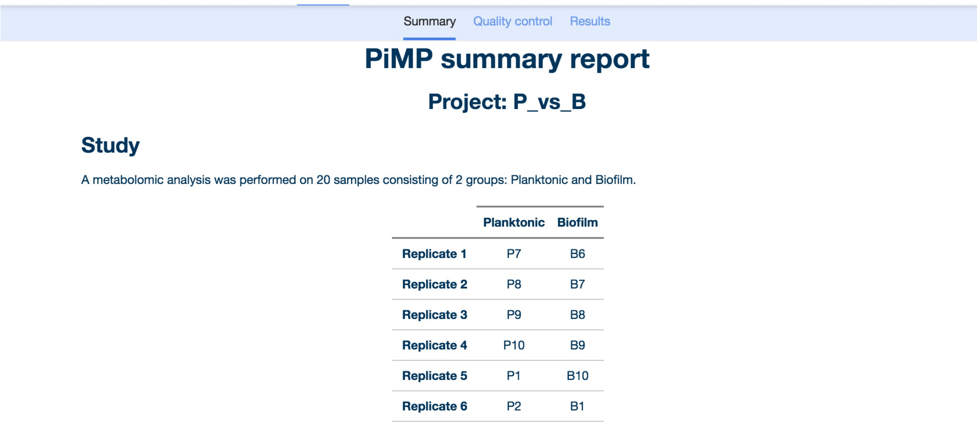
Summary Quality control Results

PiMP summary report

Project: P_vs_B

Study

A metabolomic analysis was performed on 20 samples consisting of 2 groups: Planktonic and Biofilm.

	Planktonic	Biofilm
Replicate 1	P7	B6
Replicate 2	P8	B7
Replicate 3	P9	B8
Replicate 4	P10	B9
Replicate 5	P1	B10
Replicate 6	P2	B1

Data Processing

Data Processing describes the algorithms applied to the data, with references.

Quality control

Quality control provides a number of standard methods for assessing the quality of the data: a principal component analysis and the total ion chromatograms for the datasets.

Principal Component Analysis

PCA, or principal component analysis is a multivariate statistical method for visualizing large datasets. A good experiment is usually characterized by clear separation between groups. The graph is interactive, and datapoints may be hidden or zoomed in on.

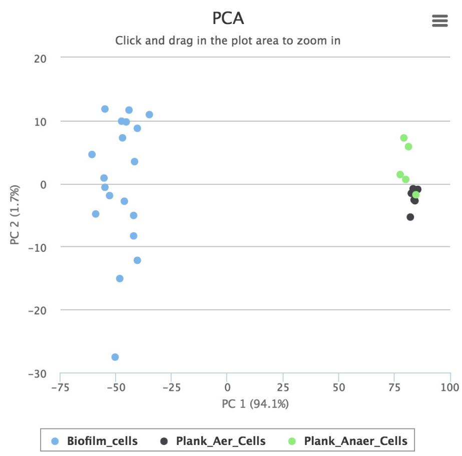

Total Ion Chromatograms

Total ion chromatograms give an overview of the total detected masses from the instrument. In a good experiment, samples should overlay to a large extent in the same group, as in the example below. Often a large broad peak is visible towards the end of the chromatogram: this is usually a consequence of salty samples using HILIC chromatography.

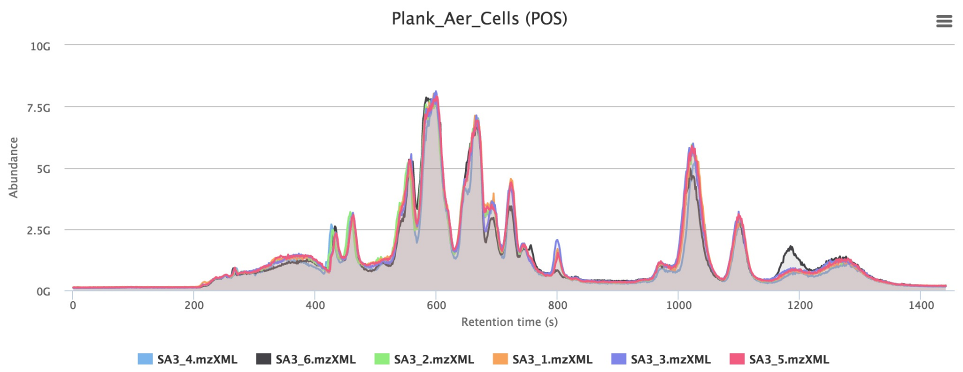

Results

Summary Page

Results provides a summary of the key findings from the data for each comparison selected by the user. Data from identified (matched by retention time and mass to a standard) compounds are shown separately from annotated (assigned putatively on the basis of mass) compounds. Only significantly changing compounds are listed here for each group. Peaks are matched by mass by seeing if the peak is within the user definable (in the Change Parameters tab) ppm range and are matched by RT by seeing if the peak is within a user definable percentage of the standard compound.

Biofilm_cells / Plank_Aer_Cells

93 identified compounds are significantly changing. The 10 most significant hits are displayed below.

Compound	Peak	Log FC	P-Value	Adj. P-Value	Biofilm_cells intensity	Plank_Aer_Cells intensity	Intensity
**Acetylcysteine**<br>**acetylcysteine**	3043	21.98	2.7e-42	5e-42	0	4299732	
**Cyclic AMP**<br>**3',5'-Cyclic AMP**	660	21.85	6e-57	1.4e-55	0	3797026	
**Guanosine monophosphate**<br>**GMP**	3868	21.56	9e-38	1.5e-37	0	3123723	
**Guanosine monophosphate**<br>**GMP**	415	21.49	8.9e-39	1.5e-38	0	2947303	
**L-Cysteate**	2672	21.43	1.1e-15	1.6e-15	37622	5381424	
**D-Erythrose 4-phosphate**	3526	20.89	4.6e-37	7.7e-37	0	2028585	
**4-Hydroxyphenylpyruvic acid**<br>**3-(4-Hydroxyphenyl)pyruvate**	4047	20.85	2.2e-10	2.9e-10	15007	3635473	
**Guanosine diphosphate**<br>**GDP**	3145	20.61	2.4e-51	1.1e-50	0	1615945	

Volcano Plots

Next, a volcano plot graphs fold change vs significance, such that the most significant and highest magnitude changes are in the top left and right of the graph. This graph is also interactive and the points can be clicked on to obtain more information about each peak.

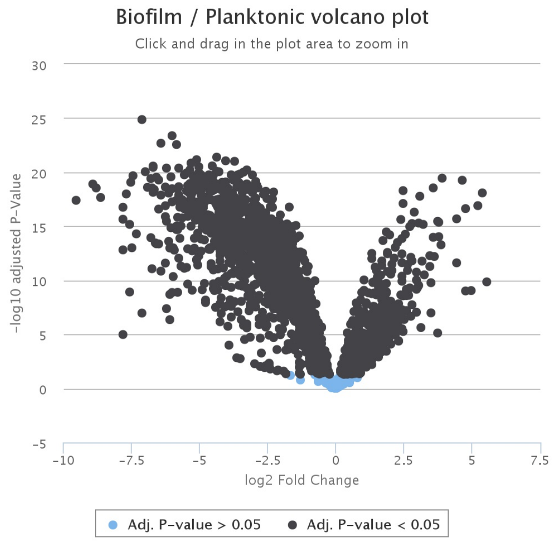

Metabolites Tab

Most researchers in metabolomics are more interested in metabolites than peaks, per se. For this reason, we decided to provide any metabolite that evidence existed for in the data. The metabolites tab summarises all the information about detected compounds, allowing the researcher to explore metabolites, pathways and their levels in different sample sets.

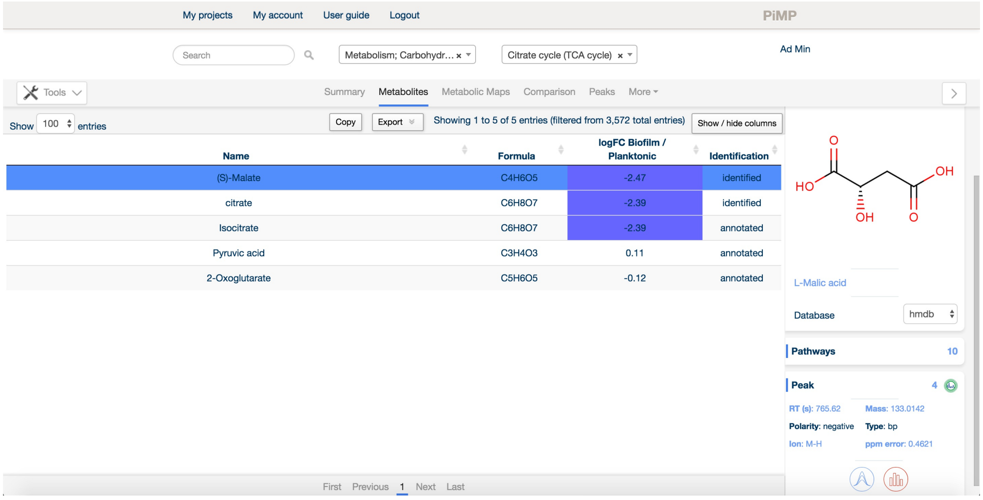

There is a free text search in the top bar that allows the researcher to narrow down on a particular compound or pathway. One can also sort on any of the columns by clicking on the title bar. Additionally, there are drop-down menus to filter the detected metabolites by pathway or superpathway.

Each experimental comparison is listed in the table next to the name and formula of each metabolite. Levels of the metabolite relative to the control condition are shown as log2 fold changes and colour coded red for upregulated and blue for downregulated. Finally, each metabolite is listed as 'annotated' or 'identified' based on the Metabolite Standards Initiative guidelines.

The 'Evidence' panel on the right of the table, activated by selecting a metabolite, provides all the evidence available for that compound, including the reference database the metabolite was found in (including the standards databases uploaded as part of the calibration samples), the structure of the compound, and any peak data associated with the compound. Simply click on the 'chromatogram' button

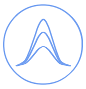

to see an interactive plot of the data.

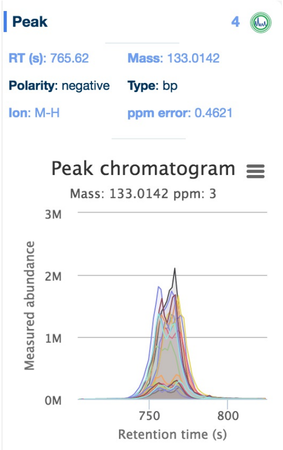

Additionally, interactive histograms of the peak can be checked below, by clicking the 'intensities' button.

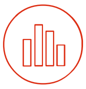

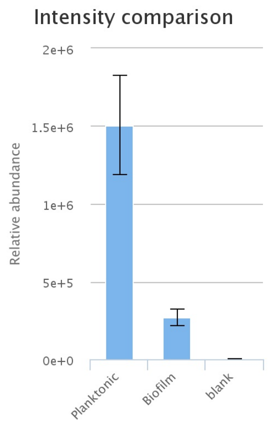

An important thing to bear in mind is the relationship of LC-MS peak to metabolite.

A single peak often can be matched to a single empirical formula, but each formula can be arranged as several structures, which are indistiguishable by mass alone. For this reason, the same peak may be assigned to multiple metabolites, all of which are displayed in the metabolites table. We have done this to avoid making any erroneous assumptions as to the priority of a metabolite assignment. For those metabolites that match by both retention time and mass, they are listed as 'identified' as that metabolite.

Each metabolite can also match several peaks, as several different structures with the same empirical formula may elute during the chromatographic separation (i.e. they will have different retention times), or may occur in multiple polarities (i.e. both positive and negative). For this reason, each 'evidence tab' may have more than one peak associated with it.

Evidence panel Tips

- Peaks matched by retention time and mass to a standard are indicated by this icon .
- The number on the top right corner of the 'peak card' indicates the number of other compounds annotated by this peak.
- A single click on the 'pathway card' gives access to the list of pathways where the metabolite is found.
- A single click on the title of the 'compound card' gives access to the compound structure.
- All cards once expanded can be collapsed for better readability
- All graphics in the evidence panel can be downloaded in different formats by clicking on in the top right corner.

Metabolic Maps Tab

The metabolic maps tab contains information about the pathways and that metabolites are assigned to. The information in this tab is derived from the Kyoto Encyclopedia of Genes and Genomes database.

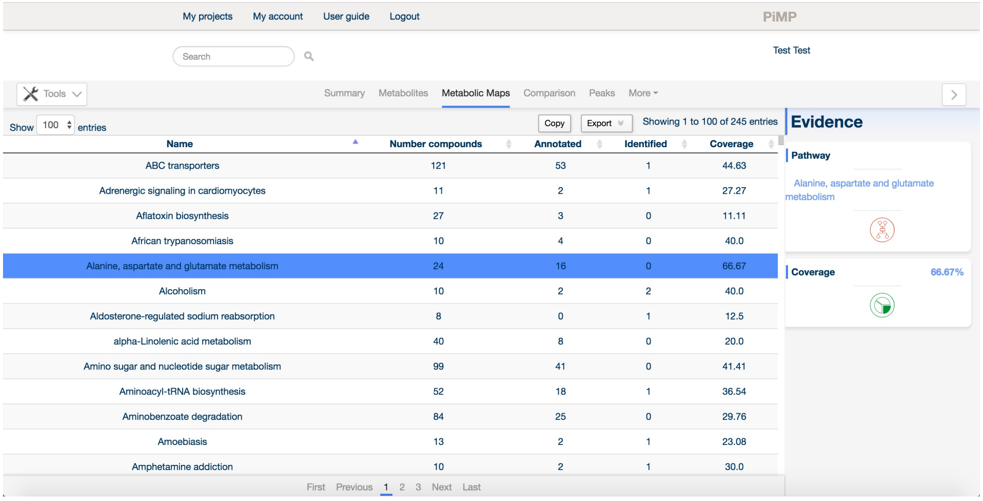

Maps are listed along with the total number of compounds in each map, the detected compounds that are listed as either annotated or identified (matched to a mass for the former, or matched to both mass and retention time for the latter), along with a coverage score that describes how much of the pathway has been detected overall.

The list of maps is searchable using the real time search bar at the top of the page.

Clicking on any of the map entries invokes a sidebar containing more information about that map, including the 'Kegg Map button' .

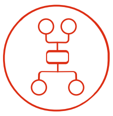

When clicked, the kegg map button will create a new window containing the Kegg map for that particular pathway, along with colour coding of the metabolites: empty circles are undetected metabolites, gold circles are identified (RT and mass matched) and grey circles are annotated metabolites.

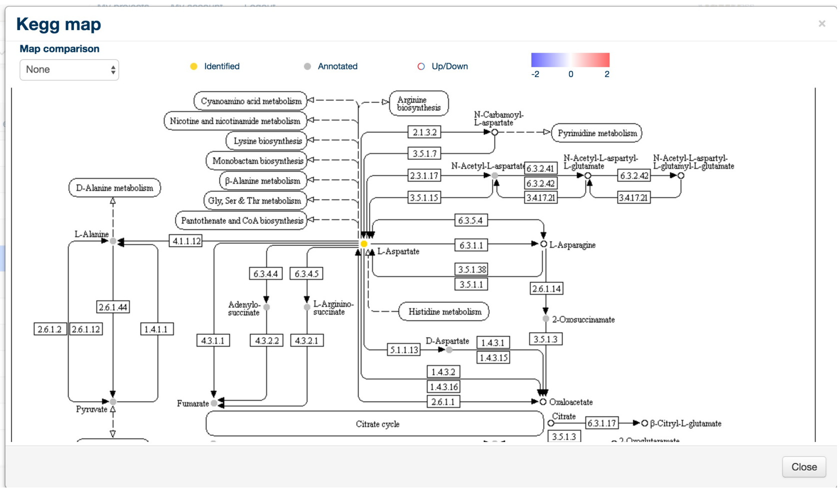

To display a quantitative overview for a specific comparison, click on the 'Map Comparison' combo box in the top left hand corner.

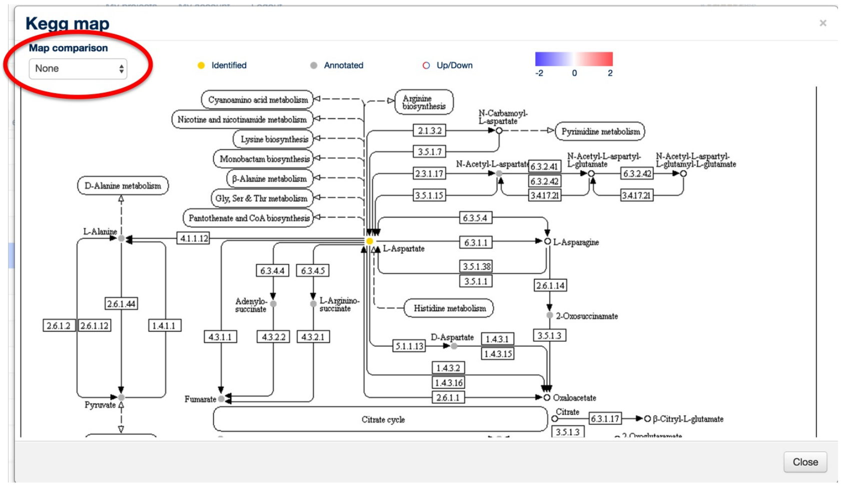

This will display the same map with rings around the individual metabolites, colour-coded according to the comparison of their levels, with red being upregulated and blue being downregulated.

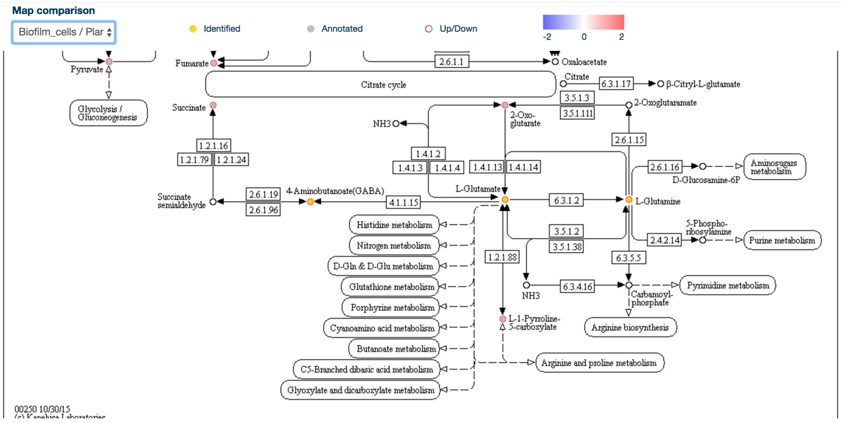

This allows a rapid overview of the data in the context of pathways, and it may be possible to identifiy metabolic chokepoints or enzymatic inhibition, if metabolites upstream an enzyme are upregulated and those downstream of the same enzyme are downregulated.

Comparisons Tab

The metabolites tab contains every metabolite for which there is evidence of its existence in the dataset. However, there are many compounds detected in a typical metabolomics analysis (especially in LC-MS) for which no known metabolite can be matched.

The majority of these are likely to be contaminants from plasticware, the atmosphere, sample handling, or (in the case of clinical samples) xenobiotics.

In some cases, however, interesting molecules are detected that may be previously undescribed. It may be worth taking a particular unknown compound forward for further characterisation, although this is very challenging and the resources required to fully characterise an unknown compound can be very significant.

For this reason, the comparison tab provides an overview of all differentially regulated compounds.

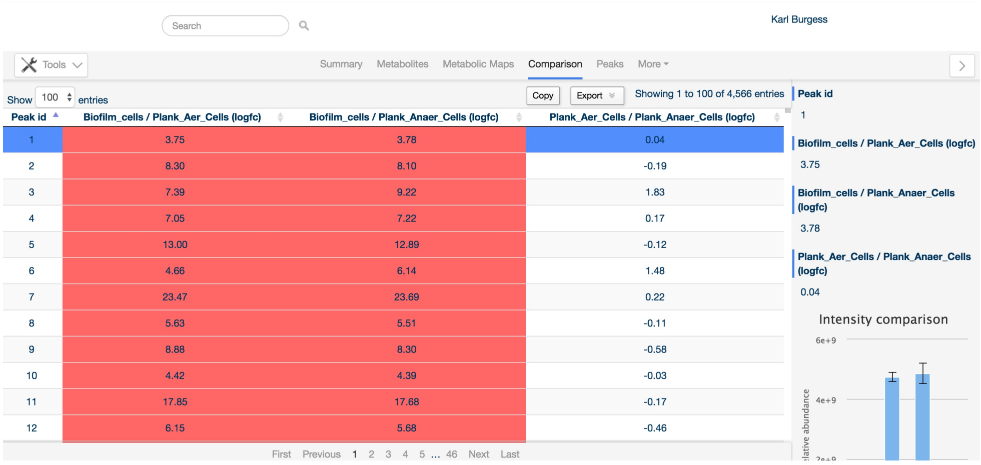

Detected compounds are listed by Peak ID (an arbitrary identifier generated by the software), and can be sorted on any of the columns, *e.g.* based on fold change of a particular comparison.

The sidebar provides detailed information about the peak of interest, including the levels of the compound in the same way as the metabolite tab. Additionally, any annotation determined for the peak is listed at the bottom of the sidebar.

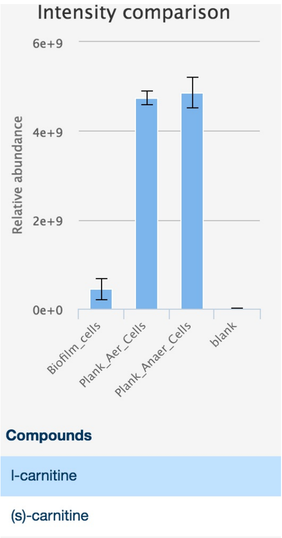

Peaks Tab

The peaks tab provide an overview of the 'raw data'. Detected features (following processing and filtering) in the LC-MS data are displayed here.

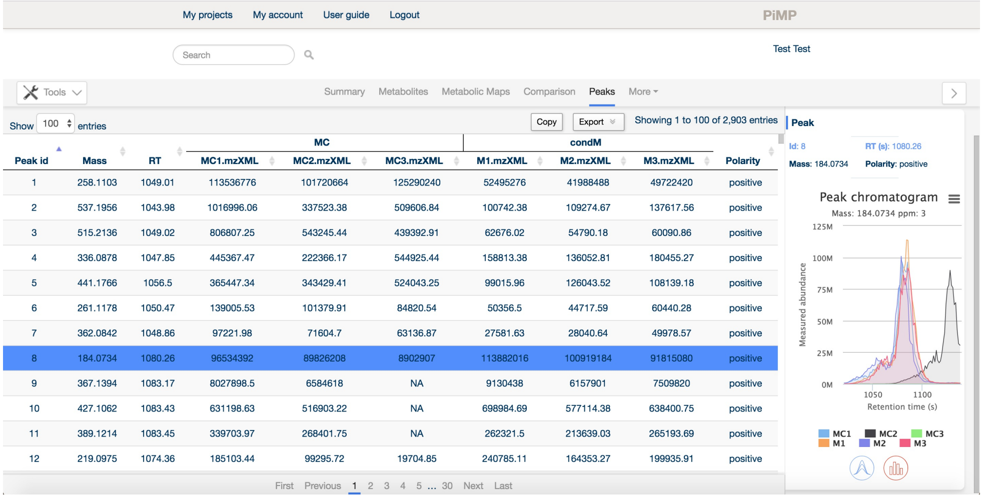

Similar to the other tabs, the panel is split into a main window and a sidebar providing detailed information about a specific peak. In the main window, the peak ID, mass and retention time are listed, along with the intensities of each peak in each sample, arranged in groups according to the comparison factors selected in the experimental definition.

The sidebar contains basic information about the peak, the intensities in each sample as an interactive histogram, and the peak chromatogram.

More tabs

The more tab provides a drop down menu of each comparison. This provides the statistical data for a specific comparison, as well as the typical sidebar providing detailed information about each peak.

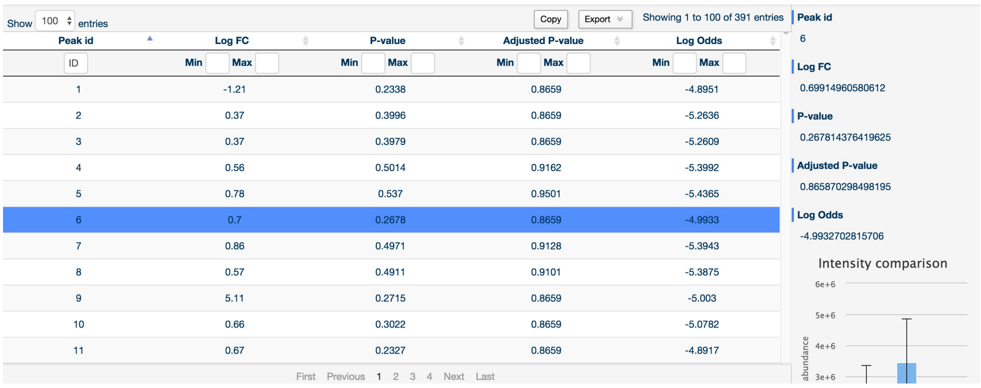

You can sort and filter by log fold change, raw p-value, Benjamini and Hochberg corrected p-value and log odds by using the two text boxes at the top of each column (left being the minimum filter and right being the maximum filter).

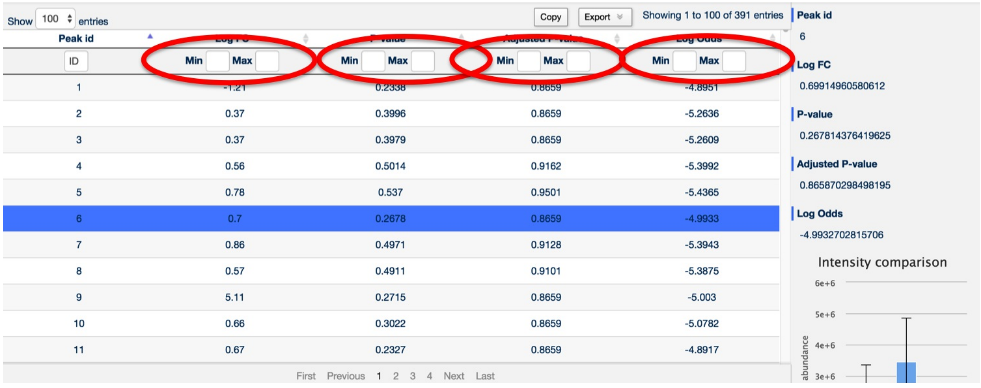

It's also possible to search for any peak containing a specific number by using the Peak ID search box at the top of the Peak ID column.

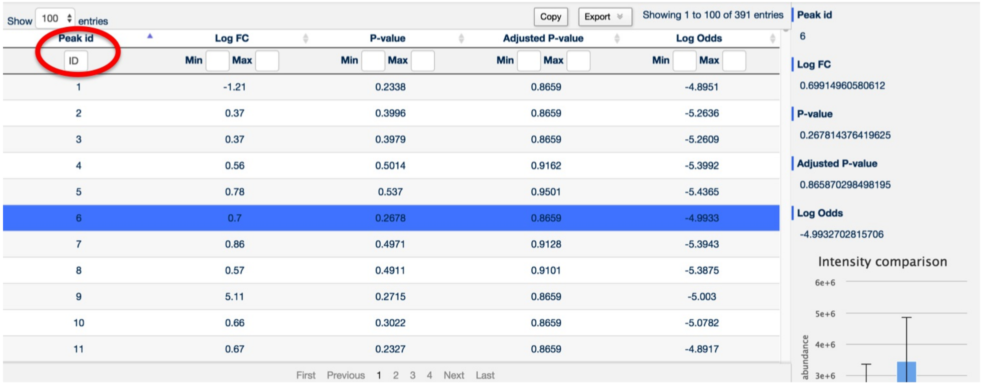

Network analysis

PiMP provides basic network analysis (of identified metabolite only) using MetExploreViz plugin. In order to start a network analysis, click on the "Network analysis" button on the left side tools menu. A popup window will appear allowing you to select the biosource and pathways to map your data on. Please note that this plugin is still at its early stage and will offer more mapping options in the future.

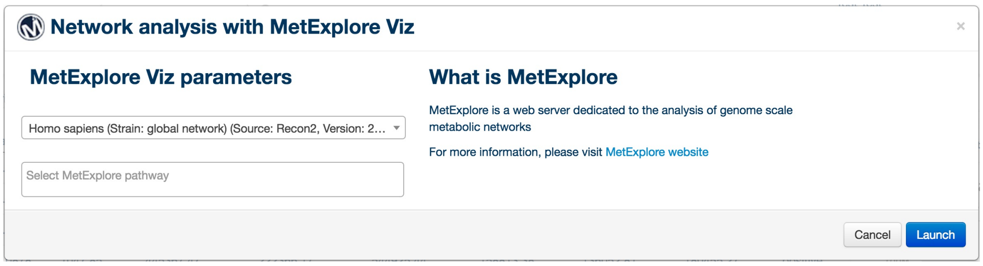

Once the biosource is selected, the drop-down pathway selection is populated, two numbers appear next to each pathway to inform on the number of metabolite in the pathway and the number of metabolite match between brackets. Press the "Launch" button to launch the tool.

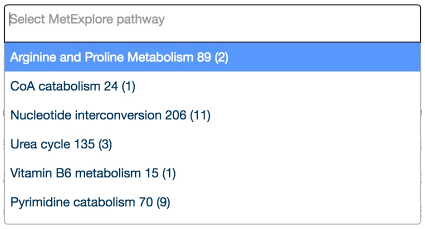

Once launched, a new tab displaying the network appears in the data environment. Several tools are available in order analyse the network and the network iteself can be exported in different format for further analysis and presentation purposes. For more information on how to use MetExploreViz please read the feature section of the documentation.

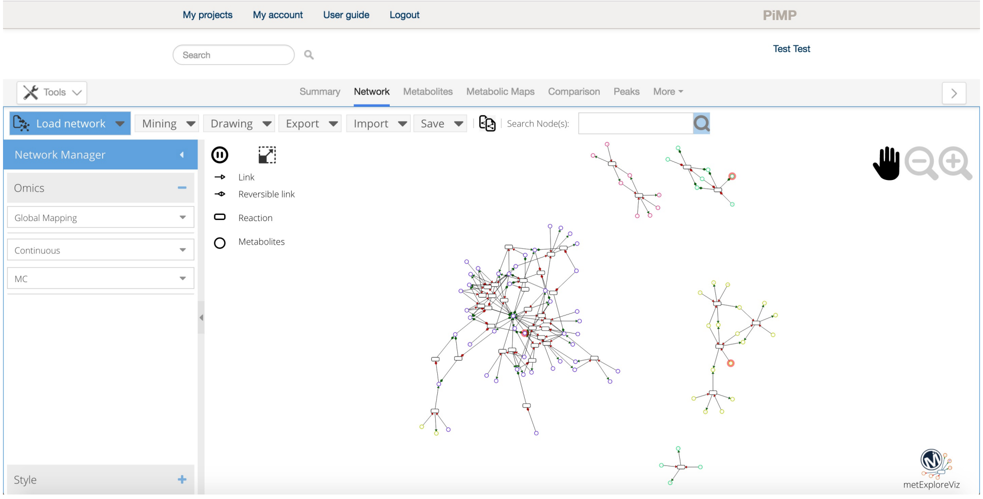

> *Network analysis Tips*
>
> - You can select as many pathway as necessary when you create your network, however if you are working on big networks (+1000 nodes) we advise to export it and analyse it within a dedicated tool such as cytoscape or directly using MetExplore.
> - The intensity data is directly accessible from the network visualisation tool, just select a condition on the left side panel to overlay the values as color scale on the network.
> - Click on the export button to download high quality images of your network for your presentations and papers.
> - Use the copy network feature 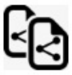 to easily compare the network under several conditions.

Preference

You can access the preference panel in order to control the chromatogram and bar charts. The preference panel can be accessed from the "tools" menu on the left side of the window.

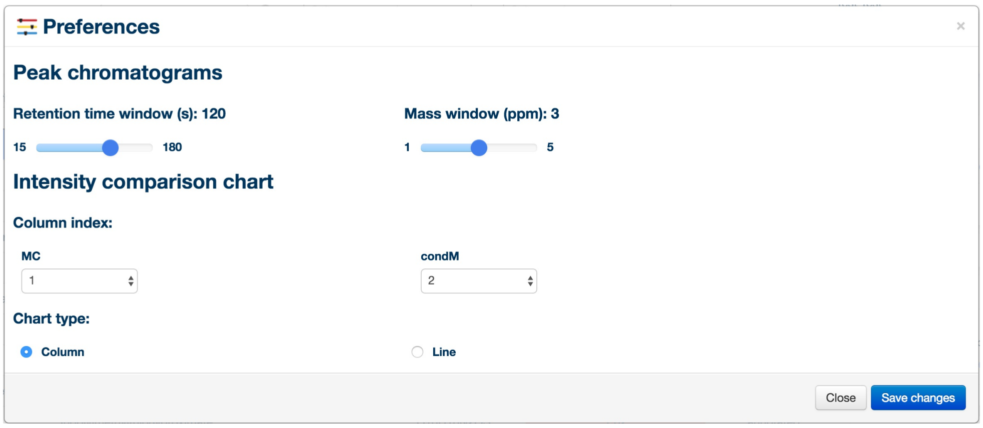

> *Preference Tips*
>
> - Looking at time course data? Select the line chart otpion and reorder your conditions to display a trend plot.

Finally

We hope that this provides you with enough information to get started with PiMP. We welcome any bug reports, suggestions and comments. Good luck with PiMP!
